# Enhanced Terahertz Fingerprint Sensing Mechanism Study of Tiny Molecules Based on Tunable Spoof Surface Plasmon Polaritons on Composite Periodic Groove Structures

**DOI:** 10.3390/s23052496

**Published:** 2023-02-23

**Authors:** Ruiqi Zhao, Yu Feng, Haotian Ling, Xudong Zou, Meng Wang, Guizhen Lu

**Affiliations:** 1 Shandong Key Laboratory of Low-Altitude Airspace Surveillance Network Technology, QILU Aerospace Information Research Institute (AIR), Chinese Academy of Sciences (CAS), Jinan 250132, China; 2 State Key Laboratory of Transducer Technology, Aerospace Information Research Institute (AIR), Chinese Academy of Sciences (CAS), Beijing 100190, China; 3 School of Integrated Circuit Science and Engineering, Tianjin University of Technology, Tianjin 300384, China; 4 State Key Laboratory of Media Convergence and Communication, Communication University of China (CUC), Beijing 100039, China

**Keywords:** THz spectroscopy, enhanced absorption, composite periodic groove structure, surface plasmon resonance, THz biosensing

## Abstract

Highly sensitive detection of enhanced terahertz (THz) fingerprint absorption spectrum of trace-amount tiny molecules is essential for biosensing. THz surface plasmon resonance (SPR) sensors based on Otto prism-coupled attenuated total reflection (OPC-ATR) configuration have been recognized as a promising technology in biomedical detection applications. However, THz-SPR sensors based on the traditional OPC-ATR configuration have long been associated with low sensitivity, poor tunability, low refractive index resolution, large sample consumption, and lack of fingerprint analysis. Here, we propose an enhanced tunable high-sensitivity and trace-amount THz-SPR biosensor based on a composite periodic groove structure (CPGS). The elaborate geometric design of the spoof surface plasmon polaritons (SSPPs) metasurface increases the number of electromagnetic hot spots on the surface of the CPGS, improves the near-field enhancement effect of SSPPs, and enhances the interaction between THz wave and the sample. The results show that the sensitivity (S), figure of merit (FOM) and Q-factor (Q) can be increased to 6.55 THz/RIU, 4234.06 1/RIU and 629.28, respectively, when the refractive index range of the sample to measure is between 1 and 1.05 with the resolution $1.54\times 10^{-5}$ RIU. Moreover, by making use of the high structural tunability of CPGS, the best sensitivity (SPR frequency shift) can be obtained when the resonant frequency of the metamaterial approaches the biological molecule oscillation. These advantages make CPGS a strong candidate for the high-sensitivity detection of trace-amount biochemical samples.

## 1. Introduction

Terahertz (THz) electromagnetic wave detection (100 GHz∼10 THz), located between the microwave and infrared bands, is considered an important topic in interdisciplinary research benefiting from its many unique properties [1,2]. The THz region covers the intramolecular vibrational-rotational energy levels of many compounds, such as hydrogen-bonding forces, van der Waals forces, rotation of dipole, lattice vibrations, and some intramolecular collective skeleton vibrations. Being a form of non-ionizing radiation, THz waves will not damage the internal structure of biomolecules during detection. Furthermore, many biomolecules have spectral signatures in the THz band, making THz wave detection applicable for variable analytes. Since THz waves exhibit good absorption selectivity on water, biomolecules and non-polar compounds, THz detection technology bears great potential in the field of biomedicine and pharmaceuticals [3,4,5,6,7].

The literature on the sensing mechanism of THz spoof surface plasmon polaritons (SSPPs) provides a technical basis for the application of THz technology in the field of substance detection and biosensing, which will directly accelerate the process of concretization and practical application of THz biosensing [8,9,10,11,12,13,14,15,16]. The concept of SSPPs is derived from surface plasmon polaritons (SPPs), a surface electromagnetic mode that confines electromagnetic energy to the subwavelength region near the metal–medium interface, and whose origins can be traced back to Wood’s discovery in 1902 of anomalous light–dark bands patterns in reflected light from mirrors covered on diffraction gratings [17,18]. SPPs originate from the coupling between the collective oscillation of free electrons at the metal–dielectric interface and the photons, which are common in the optical regime. However, the electromagnetic properties of metals in THz band are similar to that of perfect electrical conductors (PEC) and cannot support SPPs [19]. In order to extend the subwavelength-confined properties of the SPPs to the THz regime, Pendry first proposed the concept of SSPPs based on the design of a structural surface, such as conventional periodic single groove structure (CPSGS) in 2004 [20,21]. SSPPs bear similar dispersion characteristics to SPPs [22]. Therefore, the dispersion properties of SSPPs can be tuned by designing fold-like structures with different geometric morphologies and structural parameters, which have a wide range of applications in biomedicine, environmental monitoring, materials science, sub-wavelength communication and other fields [23,24,25,26,27,28,29,30,31].

As an emerging sensing technology, advanced THz biochemical detection technology enabled by SSPPs metamaterials opens up a new technical path for biochemical sensing [32]. At present, more and more outstanding interdisciplinary researchers and research institutions are engaged in the research of THz-SPR sensing based on Otto prism-coupled attenuated total reflection (OPC-ATR) configuration edging towards practical applications [33,34,35,36,37,38,39]. For example, Ng et al. studied the relationship between spectral shift of SSPPs excitation on CPSGS and refractive index by THz time-domain spectroscopy (THz-TDS) [9,40]. Yao and Zhong proposed a CPSGS-based high-order mode of SSPPs for ultrasensitive THz refractive index sensing. Zhang et al. analyzed the dependence of the resonance angle of SSPPs based on CPSGS on the change in refractive index [41,42]. Recently, Zhang and Han demonstrated that higher sensitivity can be achieved using the slanted groove in CPSGS, providing a new method for terahertz refractive index sensing [43]. The results from Chen et al. based on the CPSGS sensing device show that the sensitivity is higher when the metal grooves of a sample are filled and the semi-infinite space above the metal grooves than that of samples with only the metal grooves filled, that is, there is a trade-off between sample consumption and sensitivity [44,45]. Zhao et al. recently modified the momentum-matching condition of SSPPs on CPSGS and designed a trapezoidal double-groove structure to enhance sensor sensitivity, reaching values as high as 3.57 THz/RIU and 2.44 THz/RIU, respectively, [37,46]. In addition to the traditional fingerprint-free analysis method, Li et al. enhanced the sensing performance of trace fingerprint samples based on a new sensing mechanism combined with the meta-surface structure of SSPPs [47,48]. As mentioned in the above literature, THz-SPR biosensor technology has developed rapidly, but it still faces a formidable hurdle in improving sensitivity, reducing sample consumption, achieving specificity of fingerprint spectral analysis, and enhancing accuracy and stability [40,49,50]. Moreover, the absorption enhancement of SSPPs based on composite periodic metasurface structures has not yet been reported for THz-SPR sensing.

In this paper, we propose an enhanced tunable high-sensitivity and trace-amount THz-SPR biosensor based on the composite periodic groove structure (CPGS). First, the sensing performance of CPGS and CPSGS are theoretically analyzed and numerically verified by using the SSPPs dispersion curve obtained via the finite integral technology (FIT) and the reflection spectrum obtained using the finite element method (FEM), respectively. In addition, the concept of normalized electric field integral ratio (NEFIR) is introduced to explain their differences in sensing performance. Finally, as a biosensing application, we use the proposed CPGS-based sensor to conduct a detailed fingerprint spectrum analysis of a molecular sample. Compared with the OPC-ATR configuration based on CPSGS, the merits of the CPGS-based sensor proposed in this paper are as follows: (1) For the first time, CPGS is used instead of CPSGS to improve sensing performance while reducing sample consumption (benefiting from the near-field enhancement of SSPPs, the sample thickness is reduced from about λ to λ/100 and the performance indexes remain as high as: S = 6.55 THz/RIU, FOM = 4234.06 1/RIU, Q = 629.28). (2) By reasonably designing the dispersion curves of SSPPs and the sample thickness, it is ensured that the measured sample is completely within the effective region of near-field enhancement of SSPPs and the interaction between the incident terahertz waves and the sample is enhanced. (3) The sensor overcomes the limitation whereby the refractive index of the sample to be measured in the traditional OPC-ATR configuration needs to be smaller than that of the prism. (4) More tunable parameters are suitable for the wideband fingerprint absorption detection of various molecules.

## 2. Sensor Configuration and Physical Mechanism

**Figure 1:**
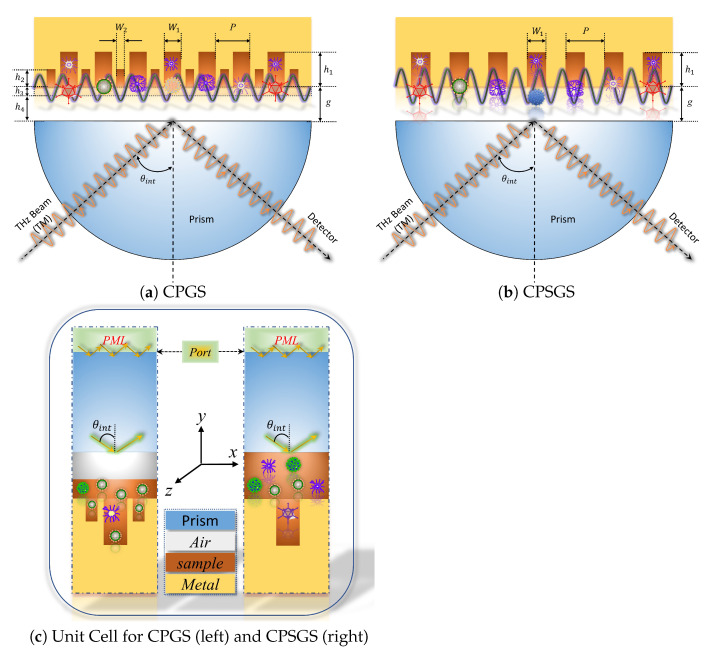
Schematic diagram of THz-SPR biological tiny molecule fingerprint sensing structure based on OTTO-ATR configuration.

The sensitivity enhancement effect of the proposed sensor is achieved by making a secondary grooving on the metal surface of the CPSGS to form a CPGS. A schematic diagram of the structure is shown in Figure 1. The period of CPGS is *P*, the width and depth of the deep groove are $W_{1}$ and $h_{1}$, and the width and depth of the shallow groove on both sides are $W_{2}$ and $h_{2}$, respectively. In order to meet the conditions of total reflection to excite the evanescent wave at the prism base and couple with SSPPs, the refractive index of the sample to be measured must be less than that of the prism layer, which severely limits the detection range of the sample in the CPSGS sensor. In the CPGS sensor, the thickness of the sample layer above the surface of the metal structure is compressed from *g* to $h_{3}$, and the space above this sample region to the prism base is replaced by air with a thickness of $h_{4}$. On the one hand, the limitation of the refractive index of the sample to be measured in CPSGS can be eliminated, and the range of detectable samples can be further expanded. At the same time, CPGS has greater design flexibility and tunability, which can realize sample detection with a wider spectral range. On the other hand, tuning $h_{3}$ and $h_{4}$ can ensure that the analyte is located in the effective region of the SSPPs enhancement field, which is essential for enhancing the interaction between the sample and SSPPs waves, and significantly increases sensing performance during real-time detection. Compared with the CPSGS, the CPGS configuration can avoid the repeated adjustment of coupling gap (*g*) to enhance the coupling efficiency between incident wave and SSPPs, especially when the coupling gap area is completely filled with samples. Because each adjustment requires the addition or reduction of samples according to different *g* values to ensure that the entire sample layer is completely filled, this will further complicate the operation and increase the measurement error. In addition, due to the absorption effect of sample molecules in liquid phase sensing will be weakened accordingly with the reduction of the sample. Therefore, another advantage offered by this sensor is that it enables liquid-phase detection of tiny molecules when the sample thickness is $h_{3}\ll g$ even in media with strong THz absorption such as water.

The physical mechanism of the proposed CPGS-based sensor is based on THz-SPR technology. As shown in Figure 1a, a TM-polarized collimated THz wave is injected into the prism with an internal incident angle $\theta_{int}$ and refracted at its base. On the one hand, when the total reflection condition $\theta_{int}>\theta_{c}$ is satisfied, the incident electromagnetic wave will be totally reflected on the prism base and generate the evanescent wave with exponential attenuation in the direction perpendicular to the interface. On the other hand, the free electrons on the CPGS-based metal surface excited by the incident THz wave will produce SSPPs that decay along the direction perpendicular to the periodic array. When the momentum of the incident THz wave matches that of the SSPPs, the so-called SPR phenomenon occurs, where a sharp dip appears in the reflected spectrum. In this way, the incident energy will be coupled into the highly localized SSPPs wave on the periodic array surface to achieve significant THz near-field enhancement effect in the subwavelength region of the array surface, thus enhancing the interaction between THz wave and the sample. By scanning the change in resonant frequency of the reflected THz wave in the broadband range covering the entire vibration absorption spectrum of the target molecule, the THz fingerprint of the molecular sample can be obtained, so as to indirectly identify the change of molecular type or composition of the measured sample. Essentially, THz-SPR sensor is a response to changes in electromagnetic parameters (refractive index, permittivity) of the sample to be measured in the THz band.

Considering the influence of the sample refractive index in CPSGS based on OPC-ATR, the excitation of SPR must meet the wave vector matching condition as shown in Equation (1), so that the evanescent wave can be coupled into SSPPs wave. However, since the prism base is in direct contact with the air domain in CPGS, the SPR phenomenon can be excited on the surface of the periodic array only if Equation (2) is satisfied [46],

(1) $$k_{SSPPs}=k_{⫽}k_{0}\frac{n_{p}}{n_{d}}\sin\left(\theta_{int}\right)$$

(2) $$k_{SSPPs}=k_{⫽}k_{0}n_{p}\sin\left(\theta_{int}\right)$$

where, $k_{SSPPs}$ is the wave vector of SSPPs, and $k_{p}^{2}=k_{⫽}^{2}+k_{⊥}^{2}$, $k_{⫽}$ and $k_{⊥}$ are the parallel and vertical components of the wave vector of incident electromagnetic wave in the prism, respectively. $n_{p}$ and $n_{d}$ are the refractive index of the prism and the sample, respectively, and $k_{0}=\omega ⁄c$ is the wave vector in vacuum. In the case of P≪λ, the wave vector of SSPPs can be expressed as,

(3) $$k_{SSPPs}=\sqrt{\epsilon_{d}k_{0}^{2}+{\left(W_{eff}/P\right)}^{2}k_{d}^{2}\tan^{2}\left(k_{d}H_{eff}\right)}$$

(4) $$k_{d}=k_{0}\sqrt{\epsilon_{d}}\sqrt{\left(1+\delta \left(i+1\right)\right)/W_{eff}}$$

(5) $$\delta =\frac{1}{k_{0}Re\sqrt{-\epsilon_{m}}}$$

where, $\epsilon_{d}=n_{d}^{2}$ is the dielectric constant of the filling medium, $\epsilon_{m}$ is the complex dielectric constant of the metal, $W_{eff}$ and $H_{eff}$ are the equivalent groove width and depth of the CPGS, respectively. According to the above equations, the dispersion relationship of SSPPs on CPGS is completely dependent on the subwavelength periodic structure and the electromagnetic characteristics of the sample, and therefore demonstrates excellent design flexibility, as expected in THz-SPR biosensing.

## 3. Simulation Results and Discussion

In order to illustrate the superior sensing performance of our proposed CPGS sensor compared with CPSGS, we first compared its sensing performance indexes on the premise of ensuring that the geometric parameters $W_{1}$, $h_{1}$ and *P* of the two periodic structures were consistent. The geometric dimensions of unit cells of the two periodic structures are shown in Table 1. Here, we mainly illustrate the coupling efficiency between incident waves and SSPPs waves by calculating the electric field strength of SSPPs on the surface of the periodic structure and the difference between their electric field integration in the sample region and all calculation domains, because higher coupling efficiency produces better sensing performance. In order to ensure the reliability of the calculation results, we used FEM and FIT methods to calculate the SPR frequency for mutual verification throughout the research process. Finally, the reflection spectrum and dispersion curves of SSPPs on CPGS filled with different refractive index samples are calculated, and the sensing performance parameters based on this structure are obtained.

**Figure 2:**
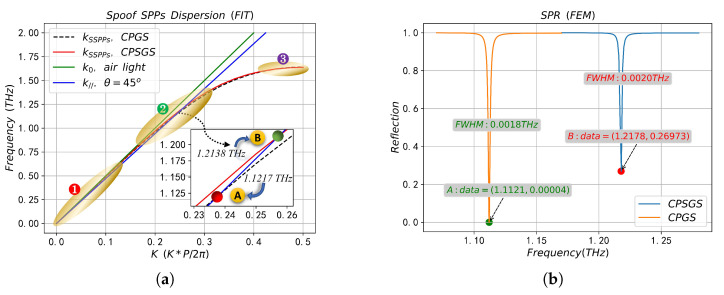
Dispersion curve (**a**) and reflection spectrum (**b**) of SSPPs on CPGS and CPSGS.

The eigenmode solver based on FIT method is used to calculate the dispersion curves of SSPPs on the surface of the two periodic structures when the filled sample is air ($n_{d}=1$), as shown in Figure 2a. When calculating the dispersion curve, the default mesh size was adopted, and the boundary conditions were set as follows: the boundary on both sides of the *x* direction was set as the periodic boundary, the *y* direction was set as $E_{t}=0$, and the *z* direction was set as $H_{t}=0$. The SPR reflection spectrum on the two periodic structures calculated by FEM method is shown in Figure 2b, when calculating the reflection spectrum, the *x* direction is the floquet periodic boundary, and the floquet periodic wave vector is $k_{F}=\left(k_{Fx},k_{Fy}\right)$, where $k_{Fx}=k_{0}n_{p}\sin\left(\theta_{int}\right)$, $k_{Fy}=0$. The top of the prism is set as the port boundary, the propagation constant at the port is set to $\beta =k_{0}n_{p}\cos\left(\theta_{int}\right)$, and the internal incidence angle $\theta_{int}$=$45^{\circ }$. A perfectly matched layer (PML) is set at the top of the computational domain to absorb the reflected wave from the *y*-direction, where the PML thickness is $\lambda_{int}⁄2$ and $\lambda_{int}$ is the wavelength of the incident electromagnetic wave. The refractive index of the prism is $n_{p}$=1.5, and the dielectric properties of metal Au are described by the drude model [51],

(6) $$\epsilon_{Au}=\epsilon_{\infty }-\frac{\omega_{p}^{2}}{\omega^{2}+j\omega \gamma }$$

where, the high frequency dielectric constant is $\epsilon_{\infty }=9.1$, the metal plasma frequency is $\omega_{p}=1.2\times 10^{16}\left(rad/s\right)$, and the electron collision frequency is $\gamma =1.2\times 10^{14}\left(rad/s\right)$. In order to ensure the convergence and accuracy of the FEM calculation results, the mesh size of different solution domains is refined by custom meshing method, in which the maximum mesh size near the surface of the metal groove is set to be $W_{2}⁄6$, and the maximum element size of the remaining mesh is $W_{1}⁄2$.

In Figure 2a, the green solid line $k_{0}$ and blue solid line *k* are wave vectors of incident light in air and prism, respectively, while the black dotted line and red solid line are dispersion curves of SSPPs on CPGS and CPSGS, respectively. The dispersion curve is below the light line, so that the SSPPs field, which decays exponentially in the direction perpendicular to the array interface, is tightly confined to the subwavelength region near the interface and propagates along the interface between the metal groove and the medium. In the low-frequency band (zone 1), the $k_{SSPPs}$ is close to $k_{0}$, indicating that SSPPs in this frequency band are quasi-static surface confinement waves, which means the coupling ability of incident light and SSPPs waves is extremely poor, and the confined ability of SSPPs fields near the metal–medium surface is extremely weak. With the increase in frequency, the dispersion curve of SSPPs gradually deviates from the light line $k_{0}$ and intersects with the medium line *k* in the middle frequency band (zone 2). The intersection point is the SPR frequency, namely point A and point B in Figure 2a. According to the phase matching condition in Equation (2), SSPPs on the periodic structure surface can only be excited near the SPR frequency point. When approaching the high frequency band of the Brillouin zone (zone 3), the dispersion curves of the two periodic structures almost completely coincide, while the difference between the dispersion curves near zone 2 is more obvious. The dispersion curve (black dashed line) of SSPPs on CPGS is located below the CPSGS (solid red line) in the zone 2, indicating that CPGS in this frequency band has better electromagnetic enhancement and confinement characteristics than SSPPs on CPSGS.

Furthermore, regarding the dispersion curve of SSPPs on CPGS, $k_{SSPPs}$ in zone 3 is successively larger than that in zone 2 and zone 1, so the field confined ability of zone 3 is successively stronger than that in zone 2 and zone 1. An optimal balance needs to be considered here. On the one hand, the higher surface-field enhancement factor in zone 3 will tightly confine the surface electromagnetic field in a smaller space than that in zone 2 and zone 1. On the other hand, the surface field-enhancement factor in zone 1 is too low compared with that in zone 2 and zone 3, which will cause more energy near the array surface to diffuse back to the region above the sample. Therefore, irrespective of whether the resonant frequency band is selected in zone 1 or zone 3, the effective overlap space volume between the analyte to be measured and the near-field enhancement region will be reduced, which will also affect the degree of resonance spectrum change of the metamaterial sensor when the analyte to be measured changes. Consequently, the sensitivity of the device is affected, so that the FOM value is also reduced. Therefore, balance zone 2 is hereafter used as the optimal frequency band for the sensor structure design.

According to the reflectance spectrum in Figure 2b, there is a clear dip in the reflectance curve at the SPR frequency points (Point A and Point B), where the minimum reflectance values are $4\times 10^{-5}$ and 0.26973, and the full width at half maximum (FWHM) of the dip in the reflection spectrum is 0.0018 THz and 0.0020 THz, respectively. It can be seen from the reflectance spectrum that the reflectivity and FWHM at the dip based on CPGS are smaller, which indicates that the absorption of this structure at the dip is significantly higher than that of CPSGS, with stronger electromagnetic confinement and enhancement characteristics and narrower reading features. The results are consistent with the theoretical analysis of dispersion curves. The resonant positions (Point A and Point B) in Figure 2a,b are almost the same, which ensures the reliability of our calculation results.

**Figure 3:**
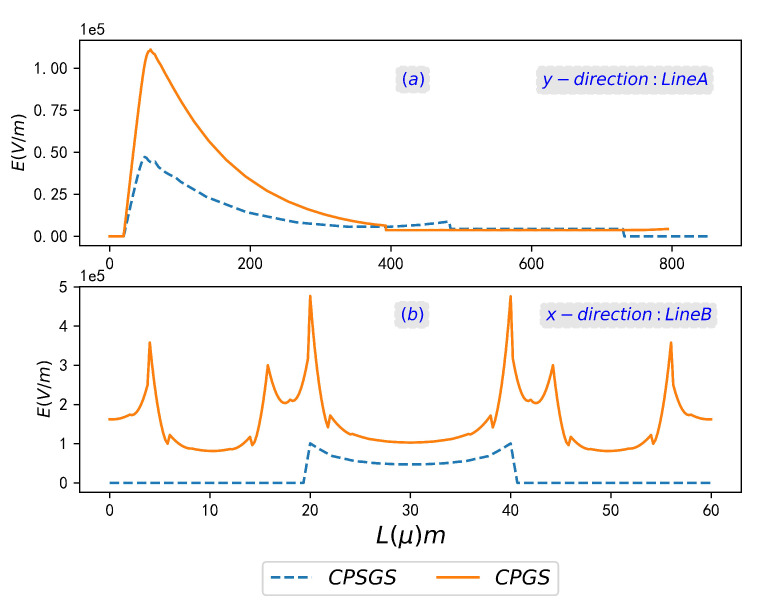
The electric field of SSPPs on CPSGS and CPGS at SPR frequency along the *y*-direction (**a**) and *x*-direction (**b**).

The above analysis qualitatively describes the stronger electromagnetic enhancement and local confinement characteristics of CPGS compared with CPSGS from the perspective of dispersion theory and SPR reflection spectrum. In order to explain the results of this theoretical analysis more intuitively, the effects of such electromagnetic enhancement and confinement are quantitatively analyzed here. We calculate the electric field in the direction perpendicular to the periodic array (Line A) and along the direction of SSPPs propagation (Line B), as shown in Figure 3. It is clear that the coupled electric field value of the CPGS surface is greater than that of CPSGS, along both Line A and Line B. Then, the normalized electric field integral ratio (NEFIR) is used to further quantitatively compare the electromagnetic enhancement effect of SSPPs on the surface of the two periodic structures. The NEFIR is described by the following formula,

(7) $$NEFIR=\frac{NEFI_{sample}}{NEFI_{all}}=\frac{<{\;\int \int \;}_{sample}\left(E_{sample}\right)dxdy>}{<{\;\int \int \;}_{all}\left(E_{all}\right)dxdy>}$$

where $E_{sample}$ and $E_{all}$ are electric field values for the sample region and all calculated domains, respectively.

**Figure 4:**
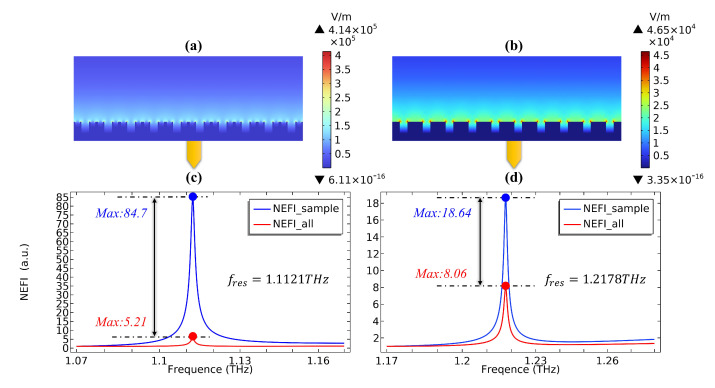
Electric field of SSPPs based on CPSGS (**a**) and CPGS (**b**), and its corresponding NEFI (**c**), (**d**) in the sample domain and the total calculation domain.

The surface electric field values of SSPPs on CPGS and CPSGS are shown in Figure 4a,b, and the corresponding maximum electric field values are $4.14\times 10^{5}$ (V/m) and $4.65\times 10^{4}$ (V/m), respectively, at a ratio of nearly 10 to 1. $NEFI_{sample}$ and $NEFI_{all}$ are the normalized electric field integrals (NEFI) of the sample region and all calculation domains, respectively. The surface electric fields of the two structures in Figure 4a,b are integrated and normalized in the above regions, where the normalization calculation is based on the integration value of the first frequency point as a reference. The integration results are shown in Figure 4c,d, where the solid blue line represents the electric field integral of the sample area, and the solid red line represents the integral of the total calculation domain. Obviously, the NEFI has a significant enhancement peak at the SPR frequency. The NEFI value of the sample region at the SPR resonant frequency in CPGS is 84.7, which is much higher than the 18.64 in CPSGS, and $NEFIR_{CPGS}\left(\left(84.7\right)⁄5.21=16.26\right)\gg NEFIR_{CPSGS}\left(\left(18.64\right)⁄8.06=2.31\right)$. A greater number of electromagnetic hot spots in CPGS leads to the concentration of more fields in the sample area compared with CPSGS, and the electromagnetic energy of the sample area accounts for a high proportion of that in the total computing domain. In other words, the field confinement ability of the SSPPs on the CPSGS is weaker than that of the CPGS. The above analysis can also be verified from Figure 4a,b. The surface electric field of CPGS is tightly confined near the interface between the metal and the sample, while the surface electric field of CPSGS is more dispersed above the sample area, which results in a significantly weakened coupling efficiency between the incident electromagnetic wave and SSPPs near the interface. Based on the above theoretical analysis and numerical verification, we can determine that the SSPPs on CPGS possesses excellent electromagnetic enhancement characteristics, and of course, better sensing performance. We will then calculate the SPR sensing performance based on this structure mainly by verifying the SPR frequency shift of different refractive index samples by FIT and FEM methods.

**Figure 5:**
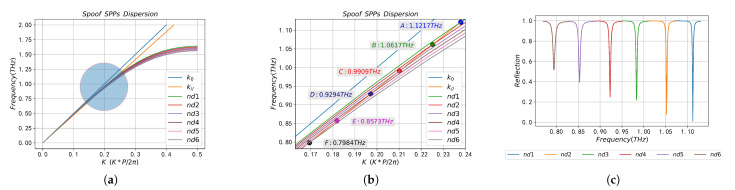
Dispersion curve corresponding to samples with different refractive indices (**a**), local magnification of dispersion curve in Figure 5a at SPR positions (**b**), SPR reflection spectrum (**c**).

The dispersion curves of SSPPs on CPGS filled with samples of different refractive indices calculated by FIT method are shown in Figure 5a. In order to clearly describe SPR frequencies corresponding to different $n_{d}$, the resonance region in Figure 5a is enlarged to Figure 5b. Here, the refractive indices of the samples were nd1 = 1, nd2 = 1.01, nd3 = 1.02, nd4 = 1.03, nd5 = 1.04, nd6 = 1.05, and the intersection point of the wave vector *k* and the dispersion curve of SSPPs with different $n_{d}$ was its corresponding SPR frequencies, namely Point A, Point B, Point C, Point D, Point E and Point F, and the corresponding frequencies were 1.1217 THz, 1.0617 THz, 0.9909 THz, 0.9294 THz, 0.8573 THz and 0.7984 THz, respectively. Obviously, with the increase in refractive index, the dispersion curve of SSPPs deviates more and more from the $k_{0}$ line, and the resonant frequency gradually redshifts. The reflectance spectra of different $n_{d}$ obtained by FEM method are shown in Figure 5c when g=343 μm, and the SPR frequencies corresponding to different $n_{d}$ are shown in Table 2, which are 1.1121 THz, 1.0522 THz, 0.9833 THz, 0.9226 THz, 0.8526 THz and 0.7939 THz, respectively. From Figure 5b,c, it can be seen that the dispersion curve reaches a good agreement with the SPR frequency of the reflection spectrum, and with the increase in $n_{d}$, the SPR frequency corresponding to the reflectance curve gradually redshifts, which is also consistent with the change of the dispersion curve.

The coupling gap is critical for the coupling efficiency between the evanescent wave and the SSPPs wave. When $n_{d}=1$, the corresponding optimal coupling gap is $g_{opt}=343$ μm, and the incident energy will be converted to the SSPPs wave on the surface of the periodic array to the maximum extent. However, with the increase in $n_{d}$, this optimal coupling state is broken, and the optimal coupling gap is greater than 343 μm. This is mainly because the penetration (attenuation) distance of the evanescent wave in the medium is,

(8) $$L_{1}=\frac{1}{k_{0}\sqrt{{\left(n_{p}\sin\left(\theta_{int}\right)\right)}^{2}-n_{d}^{2}}}$$

The attenuation distance (confinement) of SSPPs waves along the perpendicular interface direction are defined as,

(9) $$L_{2}=\frac{1}{k_{0}\sqrt{{\left(k_{SSPPs}/k_{0}\right)}^{2}-n_{d}^{2}}}$$

Therefore, $L_{1}$ and $L_{2}$ will also increase with the increase in $n_{d}$, and the attenuation intensity of evanescent wave and SSPPs wave in the medium will also be weakened correspondingly. The optimal coupling state under the initial condition is broken, results in partial reflection on the surface of the metal periodic structure, leading to the reduction of coupling efficiency. It is identified by the increasing FWHM value and the reflectivity amplitude at dip in the reflection spectrum, as shown in Figure 5c. Therefore, the distance of the coupling gap must be increased to ensure that the incident wave energy still couples perfectly into the SSPPs wave as $n_{d}$ increases.

**Figure 6:**
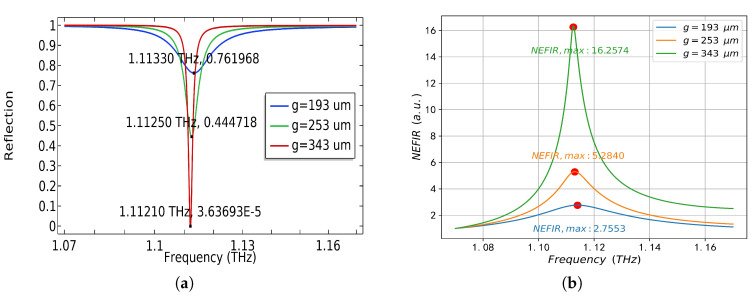
The reflection spectrum for different coupling gaps (**a**) and the corresponding NEFIR (**b**).

**Figure 7:**
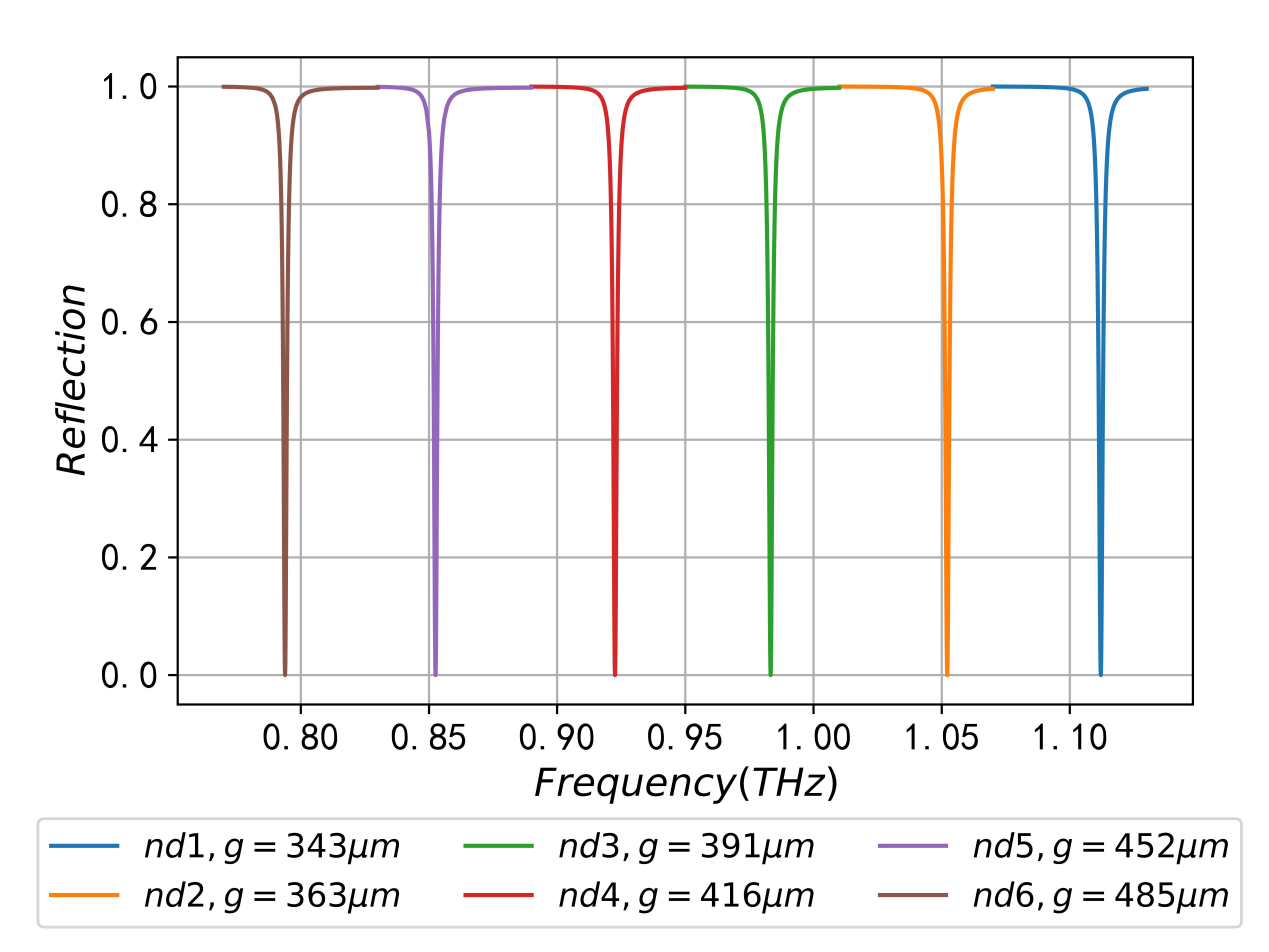
SPR reflection spectra of different sample refractive indices based on CPGS under optimal coupling conditions.

In order to illustrate the above theoretical analysis, Figure 6 shows the reflection spectrum of different coupling gaps at $n_{d}=1$ and the corresponding NEFIR of the sample domain and the total calculation domain. As can be seen from Figure 6a, with the increase in *g*, the SPR frequency in the reflection spectrum is almost unchanged, but the minimum FWHM value and reflectivity at dip is getting smaller. The absorption peak is getting sharper, which is exactly the direction expected by THz-SPR sensing. It can be seen from Figure 6b that with the increase in *g*, the NEFIR between the sample region and all calculation domains also increases. When the coupling gap is the optimal value, i.e., g=343 μm, the NEFIR reaches the maximum, about 16.26, and SSPPs wave is strictly confined on the periodic structure surface. The reflection spectrum for different refractive indexes in the condition of optimal coupling are shown in Figure 7, it can be seen that with the increase in $n_{d}$, the corresponding *g* value is increased accordingly, and the perfect coupling between the incident electromagnetic energy and SSPPs wave filled with different $n_{d}$ samples can be obtained. The comparison between Figure 5c, Figure 6a and Figure 7 shows that coupling gap does not affect the SPR frequency. Therefore, the SPR frequencies in Figure 5c and Figure 7 are consistent, and the difference is only recognized in the minimum reflectivity at dip and the change of FWHM. The sensor performance parameters obtained from the reflectance curve in Figure 7 are shown in Table 2, and it can be seen that the minimum reflectivity amplitude $R_{min}$ at dip under perfect coupling are in the order of $1\times 10^{-5}$, which represents an absorption rate of up to 99.99%.

In order to measure the performance of the proposed CPGS-based THz-SPR sensor, the frequency sensitivity (S), figure of merits (FOM), Q-factor (Q) and refractive index resolution ($\delta n_{d}$) are used for comprehensive evaluation. The calculation formula is as follows,

(10) $$S=\frac{\Delta f_{res}}{\Delta n_{d}}=\frac{f_{res,2}-f_{res,1}}{n_{d,2}-n_{d,1}}$$

(11) $$FOM=\frac{S}{FWHM}=\frac{S\times Q}{f_{res}}$$

(12) $$Q=\frac{f_{res}}{FWHM}$$

(13) $$\delta n_{d}=\frac{\delta f_{res}}{S}$$

where, $f_{res,1}$ and $f_{res,2}$ are SPR frequencies when the sample refractive index is $n_{d,1}$ and $n_{d,2}$, respectively. FWHM represents the full width at half maximum at dip of the reflection spectrum. $\delta f_{res}$ is the frequency resolution defined by the resonance FWHM or the spectral resolution of the radiation source. In conventional THz time-domain systems, $\delta f_{res}$≈ 5 GHz can be decreased to the limit of about 100 MHz [52]. The frequency sensitivity is the change of SPR frequency caused by the change of refractive index unit (RIU), which is closely related to the structure and material of the unit cell of the metamaterial resonator. FOM are used to evaluate both the resonance characteristics of the metamaterial itself and the responsiveness of the metamaterial sensor to the analyte. The Q-factor describes the quality of the SSPPs resonance and, to some extent, the near-field enhancement effect of SSPPs.

The effective combination of the sample to be measured and the metamaterial field enhancement region is also an important factor in determining the sensitivity of the metamaterial to the analyte. Only when the analyte acts in the effective region can the interaction between the analyte and the THz wave be greatly enhanced. In order to obtain the extremely high field enhancement factor, the metamaterial resonator unit is designed as a sub-wavelength composite periodic groove structure to couple the incident field to the SSPPs wave on the surface of the structure, so as to realize the near-field enhancement in a very small space. This extremely high enhancement effect usually manifests itself as a very high Q-factor in the reflection and absorption spectrum. A higher Q-factor means more sensitive sensing performance and contributes to the high resolution in spectral characterization. However, the surface enhancement field with a higher Q-factor will be confined to a smaller space, which will reduce the effective overlapping space volume between the analyte and the enhancement field region. This consequently affects the degree of change of the metamaterial sensor’s resonance spectrum when the analyte changes, which in turn affects the sensitivity of the sensor and reduces the FOM value. Therefore, from Equation (11), it can be seen that the factors (S, FOM, Q) affecting the performance of metamaterial sensors show a relationship of checks and balances, and there is a certain upper limit of improvement for metamaterial sensors that only rely on increasing Q-factors to achieve higher sensitivity. Identifying methods to reasonably balance the resonance characteristics of metamaterial, the weight between the field enhancement region and the binding mode of the analyte, such as the coincident volume, represents the key factor to further improve the performance of metamaterial biochemical sensors.

**Figure 8:**
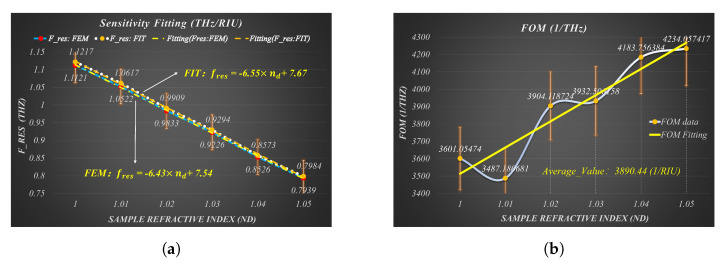
The fitting curves of Sensitivity (**a**) and FOM (**b**).

We reasonably take into account the above factors in the proposed metamaterial sensor design based on CPGS, and comprehensively improve the above sensing performance indicators by compressing the thickness of the sample layer to the effective area of near-field enhancement and repeated grooving on the basis of a single groove to form a composite groove structure. As shown by the data in Table 2 and the sensitivity and FOM fitting curves in Figure 8, the designed THz-SPR sensor has an extremely high sensitivity of 6.55 THz/RIU, an average FOM of approximately 3890.44 1/RIU, an extremely high Q-factor of 629.8, and a minimum $\delta n_{d}$ of $1.54\times 10^{-5}$ RIU. In addition, the fitting curve, the SPR frequency, and both the dispersion curve and the reflection spectrum in Figure 8a are in good agreement, which further ensures the reliability of our data calculation.

In order to illustrate the outstanding superiority of the proposed CPGS-based THz-SPR sensor in sensing performance and provide a comparison with previous works, Table 3 shows the performance indicators of the SPR sensor based on OPC-ATR configuration in the THz band. For metamaterial sensors based on CPGS, when the frequency of the incident electromagnetic wave is equivalent to the collective oscillation frequency of the free electrons in the metamaterial, the resonant electromagnetic field is strongly confined near the interface below the sub-wavelength scale, which can greatly enhance the interaction between electromagnetic waves and matter. When the TM-polarized electromagnetic wave irradiates the characteristic groove gap of CPGS, confined electromagnetic fields can be generated at these groove gaps due to the mode coupling effect between evanescent wave and SSPPs wave, which makes the electromagnetic fields highly concentrated and forms electromagnetic “hot spots”.

Compared with CPSGS, the mechanism of CPGS in enhancing THz-SPR sensing performance is described as follows: (1) The introduction of composite grooves can further reduce the plasma frequency, thereby enhancing the confinement ability of SSPPs and realizing the near-field enhancement of plasmonic; (2) Under the premise of keeping the period of the metamaterial structure unchanged, the number of electromagnetic hot spots on the surface of the periodic grooves is increased by changing the single groove based on CPSGS into the composite groove based on CPGS (to improve the space ratio of hot spots), so as to achieve electromagnetic energy concentration; (3) By adjusting the coincident volume of the sample and the SSPPs enhancement field area, the sample is completely located in the region of the enhanced field with very little space (hot spot area), thereby enhancing the interaction between the sample and SSPPs coupling near field.

## 4. A Proof of Concept of THz Tiny Molecular Fingerprint Detection

**Figure 9:**
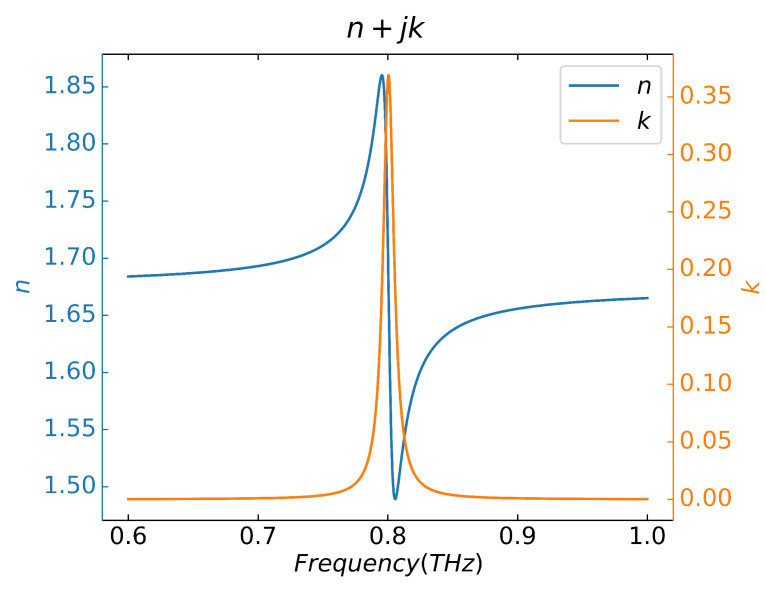
Complex refractive index of tiny biomolecules in THz band.

The theoretical analysis and numerical verification of the sensing performance of the proposed CPGS-based THz SPR sensor demonstrate ultra-high sensitivity, FOM and Q-factors, extremely small reflectivity amplitude and refractive index resolution of the sensor, which are excellent indicators for tiny biomolecular fingerprint sensing. In order to illustrate the application prospect of the designed sensor in fingerprint analysis, we will next conduct numerical simulation on the fingerprint spectrum of tiny biomolecules. Here, the complex dielectric constant of hypothetical tiny molecules is described by the Drude–Lorentz dispersion model [53],

(14) $$\epsilon \left(\omega \right)=\epsilon_{\infty }+\sum_{j=0}^{M}\frac{f_{j}\omega_{p}^{2}}{\omega_{j}^{2}-\omega^{2}+i\omega \gamma_{j}}$$

where, the high-frequency permittivity $\epsilon_{\infty }=2.8$, the oscillator strength $f_{1}=1$, the plasma frequency $\omega_{p}=0.1$ THz, the vibration frequency of the sample molecule $\omega_{1}=0.8$ THz, and the damping coefficient $\gamma_{1}=0.01$ THz, respectively. The relationship between the complex permittivity and the complex refractive index is $\tilde{n(\omega )}=n\left(\omega \right)+jk\left(\omega \right)=\sqrt{\epsilon (\omega )}$. The complex refractive index of the biomolecule described by the Drude-Lorentz dispersion model is shown in Figure 9, where the real part n(ω) of the complex refractive index is higher than that of the prism in the entire frequency band. Clearly, the excitation conditions of SSPPs in CPSGS-based OPC-ATR configuration that strictly limit the refractive index of the sample to be less than that of the prism ($n\left(\omega \right)<n_{p}$) cannot be satisfied, so the fingerprint sensing cannot be realized. However, the proposed sensor structure compresses the sample layer thickness to a much smaller width than the coupling gap, i.e., $h_{3}\ll g$, therefore the above conditions ($n\left(\omega \right)<n_{p}$) no longer needs to be met. The absorption characteristics of the sample molecules are mainly derived from the imaginary contribution of the complex refractive index, so it is not difficult to imagine that the linear shape of the intrinsic absorption spectrum should also be consistent with the imaginary part of the complex refractive index in Figure 9, which can be verified from the absorption spectrum in Figure 11a.

In order to study the sensing mechanism of SPR fingerprint spectrum of sample molecules on CPGS, on the one hand, we tuned the overlap area between sample molecules and SSPPs coupling-field enhancement region by tuning the thickness of sample layer $h_{3}$, so as to enhance the interaction between sample and SSPPs wave. On the other hand, by designing the CPGS metamaterial, the resonant frequency $f_{SSPPs}$ of its structure gradually approaching the natural resonance frequency $f_{nature}$ that can represent the tiny molecule. It can be seen from the reflectance spectrum that when the $f_{SSPPs}$ approaches the $f_{nature}$, the SPR frequency can produce a larger spectral shift after filling the sample molecules, while the spectral shift is not obvious when the fingerprint molecules are detected by sensors designed with other structural parameters.

**Figure 10:**
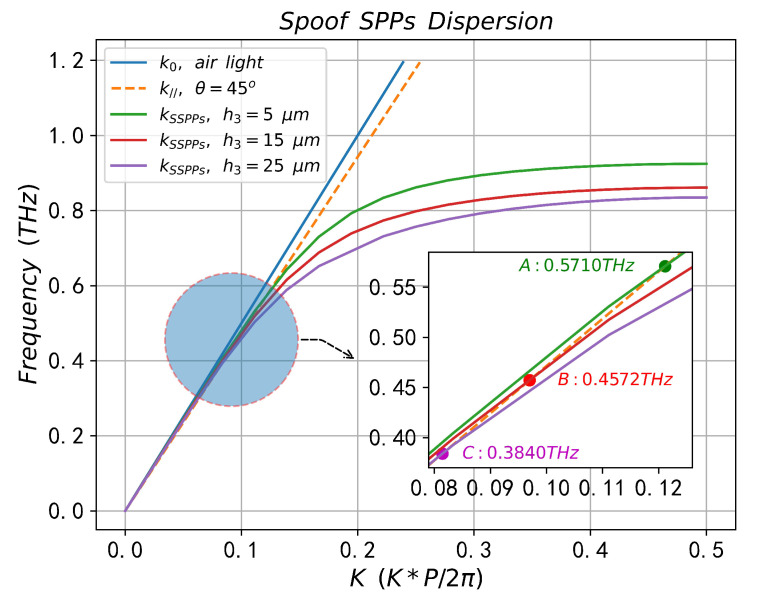
The dispersions of SSPPs with different sample thicknesses ($h_{3}$ = 5 μm, 15 μm, 25 μm).

**Figure 11:**
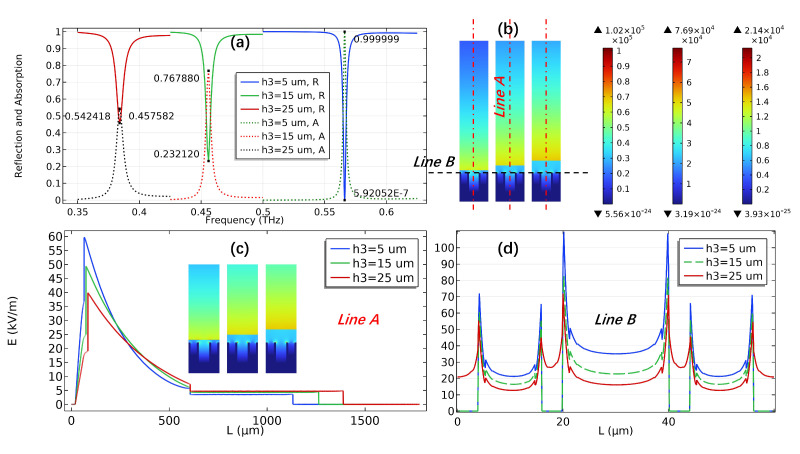
Reflection and absorption spectra (**a**), surface electric field distribution (**b**) and electric field diagrams along Line A (**c**) and Line B (**d**) of SSPPs with different sample thicknesses ($h_{3}$ = 5 μm, 15 μm, 25 μm).

The influence of sample thickness on the interaction between sample molecules and SSPPs waves is shown in Figure 10 and Figure 11. The corresponding geometric parameters are: $h_{1}$ = 40 μm, $h_{2}$ = 25 μm, $W_{1}$ = 20 μm, $W_{2}$ = 12 μm, *P* = 60 μm. Figure 10 shows the dispersion curves of SSPPs with different sample thicknesses and Point A, Point B and Point C are the corresponding SPR frequencies when $h_{3}$ = 5 μm, 15 μm and 25 μm, respectively, which is in good agreement with the SPR position in the reflectance spectrum of Figure 11a. The reflection (absorption) spectra, electric field distribution and electric field diagrams along the directions of Line A and Line B with different sample thicknesses are shown in Figure 11a–d, respectively. According to Figure 11a, when $h_{3}$ = 5 μm, 15 μm and 25μm, the minimum reflectivity at the dips are $5.92\times 10^{-7}$, 0.2321 and 0.4576, respectively, and the corresponding absorption rates are 99.99%, 76.79% and 54.24%, respectively, and the SPR frequencies were 0.5662 THz, 0.4560 THz and 0.3840 THz, respectively. Figure 11b shows the electric field values corresponding to $h_{3}$ = 5 μm, 15 μm and 25 μm from left to right, which are $1.02\times 10^{5}$ (V/m), $9.77\times 10^{4}$ (V/m), and $8.4\times 10^{4}$ (V/m), respectively. Figure 11b–d can clearly explain the difference in reflectance at dips in Figure 11a, when $h_{3}$ = 5 μm, the sample molecular layer is in the region where SSPPs waves have the strongest confinement effect on the surface of the periodic structure, which means that the interaction between sample molecules and SSPPs waves (absorption) is the strongest.

**Figure 12:**
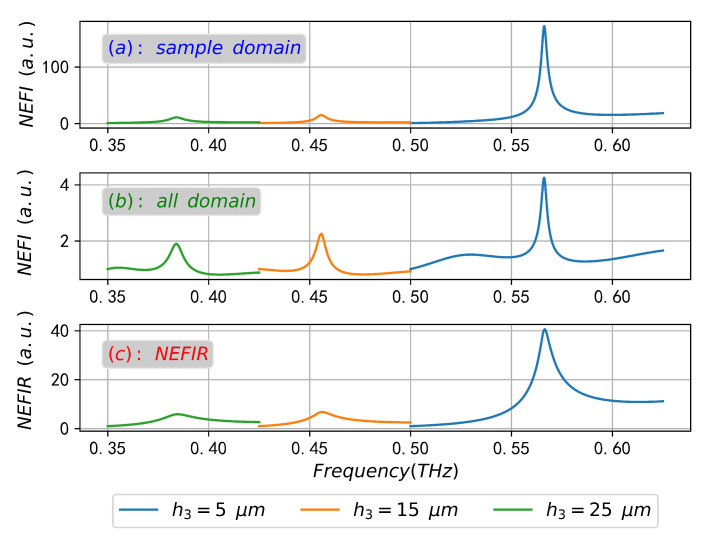
The NEFI for sample area (**a**) and all computational domains (**b**), and the NEFIR (**c**).

Figure 12 also compares the contribution of sample thickness to enhance the interaction between sample molecules and the coupling field from the perspective of NEFIR. It is clear that NEFIR of $h_{3}$ = 5 μm is much larger than that of other sample thicknesses, and more fields are confined to the area near the surface of periodic structure. However, in the case of other sample thicknesses, some fields are reflected back to (diffused in) the region above the sample, which further reduces the contact volume and weakens the interaction between the sample molecules and the coupling field, and results in weaker absorption.

**Figure 13:**
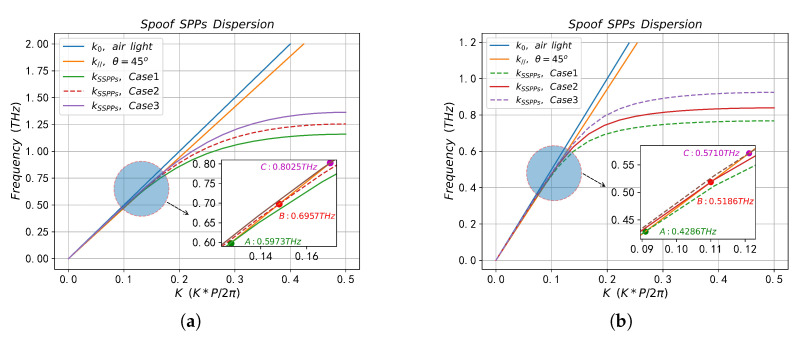
SSPPs dispersion curves corresponding to the different resonant structures (Case1, Case2 and Case3) without sample (**a**) in Table 4 and with sample (**b**) in Table 5.

**Figure 14:**
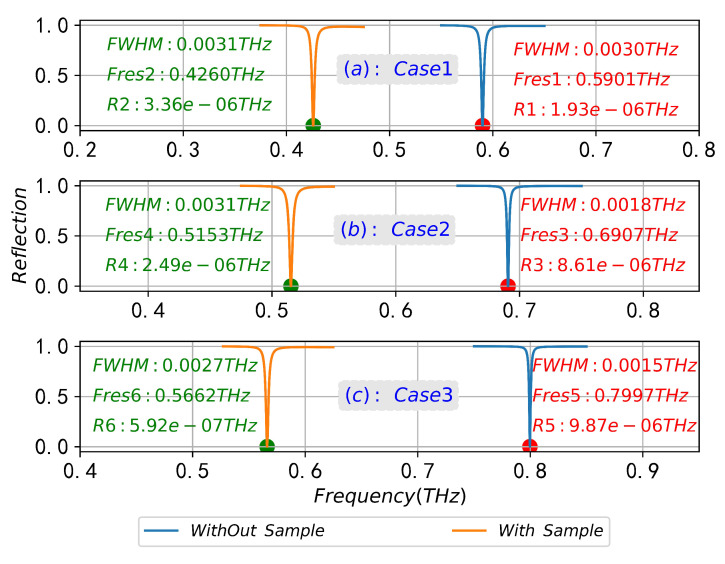
SPR reflection spectrum corresponding to the different resonant structures (Case1, Case2 and Case3) without sample (**a**) in Table 4 and with sample (**b**) in Table 5.

**Figure 15:**
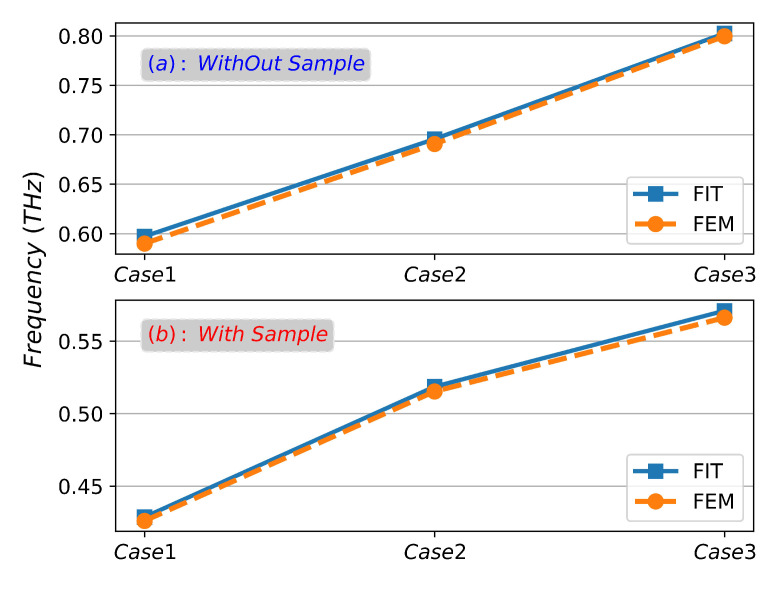
The SPR frequency of SSPPs on CPGS with different $f_{SSPPs}$.

Since the natural resonance frequency of this biomolecule is $f_{nature}$ = 0.8 THz, we designed three CPGS-based metamaterial sensors with different resonant frequencies ($f_{SSPPs}$ = 0.6 THz, 0.7 THz and 0.8 THz) under the optimal sample thickness $h_{3}$ = 5 μm, so that the $f_{SSPPs}$ gradually approached $f_{nature}$ to study the interaction between the sample and SSPPs field and its SPR frequency shift characteristics, detailed geometric parameters are shown in Table 4 (no sample) and Table 5 (with sample). Figure 13a,b show the dispersion curves of SSPPs in the absence of samples and with samples, and Figure 14a–c are the reflection spectra of SSPPs on the surface of metamaterial structures with different $f_{SSPPs}$ under the optimal coupling conditions ($g=g_{opt}$) in the absence of samples and with samples. It can be seen from Figure 13a that the SPR frequency (Point A, Point B and Point C) of the designed CPGS sensor without samples is basically consistent with the expected $f_{SSPPs}$ (0.6 THz, 0.7 THz and 0.8 THz), which is very important and provides a reference for our next step of calculating the spectrum shift. From Figure 13b and Figure 14, it can be seen that when fingerprinted samples are present, the SPR frequency undergoes a greater redshift in the spectrum as the $f_{SSPPs}$ gradually approaches the $f_{nature}$. The reflectivity at dip and the FWHM is minimal, and the minimum reflectivity ratio with and without samples exceeds an order of magnitude ($R_{5}/R_{6}\gg 16$). However, when the sensors designed with other structural parameters are used to detect the fingerprint molecules, the spectral redshift, FWHM and the reflection amplitude at dip do not change significantly. Finally, Figure 15a,b show the SPR frequencies of different metamaterial structures with and without samples calculated by FIT method and FEM method, respectively, and the curves are in good agreement, which mutually verify the reliability of the previous numerical results and theoretical analysis.

## 5. Conclusions

In summary, in order to improve the problems of poor tunability, low sensitivity, poor refractive-index resolution, significant absorption of background water molecules resulting from large sample consumption, inability to achieve trace detection, and lack of fingerprint spectrum analysis based on the CPSGS, we propose an enhanced THz-SPR biosensor based on CPGS. In order to enhance the interaction between the sample and SSPPs waves, and then comprehensively improve the sensing performance of the THz-SPR sensor, first, multiple grooves are introduced into the proposed sensor to form CPGS on the basis of CPSGS, thus increasing the number of electromagnetic hot spots on the surface of the periodic structure. Second, the CPGS-based sample consumption is reduced by a factor of about 100, and its normalized electric field integration ratio (NEFIR) is about 7 times that of the CPSGS-based sample, while the sensitivity and FOM are improved by 83.47% and 6.75%, respectively, as compared to optimal values reported in the literature. Finally, the FIT method and FEM method mutually verified the accuracy of SPR frequency points and ensured reliability of the results. The CPGS metamaterial sensor demonstrates good tunability via the gradual tuning of geometric parameters to drive the resonance frequency ($f_{SSPPs}$) of the metamaterial to match that of the biological sample molecules’ natural resonance frequency ($f_{nature}$). It can be seen from the reflection spectrum that a larger spectral shift in SPR frequency occurs, while the spectral shift is not significant when the fingerprint molecule is detected by the sensors designed with other structural parameters. Therefore, benefiting from the more tunable parameters of CPGS, tuning $f_{SSPPs}$ to near $f_{nature}$ can greatly improve its sensing performance, which is extremely important for the detection of highly sensitive trace-amount biochemical samples in future applications.

## Figures and Tables

**Table 1:** The geometric dimensions of unit cells.

	Parameters (μm)	$W_{1}$	$W_{2}$	$h_{1}$	$h_{2}$	$h_{3}$	$h_{4}$	*g*	*P*
Cell									
CPGS		20	12	30	14	105	$g-h_{3}$	343	60
CPSGS		20		30				343	60

**Table 2:** Performance index parameters of THz-SPR sensor based on CPGS.

$n_{d}$	$f_{\mathrm{res}}$ (THz)	FWHM (THz)	$R_{\mathrm{res}}$	*S* (THz)	FOM (1/THz)	*Q*	$\delta n_{d}$ (RIU)
1.00	1.1121	$1.77\times 10^{-3}$	$3.64\times 10^{-5}$	6.36	3601.05	629.28	$1.57\times 10^{-5}$
1.01	1.0522	$1.72\times 10^{-3}$	$3.92\times 10^{-5}$	6.00	3487.18	612.56	$1.67\times 10^{-5}$
1.02	0.9833	$1.65\times 10^{-3}$	$3.37\times 10^{-6}$	6.44	3904.12	596.11	$1.56\times 10^{-5}$
1.03	0.9226	$1.61\times 10^{-3}$	$2.31\times 10^{-5}$	6.32	3932.51	574.37	$1.58\times 10^{-5}$
1.04	0.8526	$1.55\times 10^{-3}$	$1.77\times 10^{-5}$	6.49	4183.76	549.84	$1.54\times 10^{-5}$
1.05	0.7939	$1.50\times 10^{-3}$	$1.44\times 10^{-5}$	6.36	4234.06	528.19	$1.57\times 10^{-5}$

**Table 3:** Comparison of the sensing performance of THz-SPR sensors based on CPGS and CPSGS.

Reference	Method	Frequency (THz)	$R_{\mathrm{res}}$	*S* (THz)	FOM (1/THz)	*Q*	$\delta n_{d}$ (RIU)
[9], 2013	CPSGS	1.7100	-	0.52	49	4.6	-
[41], 2014	CPSGS	1.9100	-	2.27	262	-	$4\times 10^{-3}$
[44], 2018	CPSGS	0.2650	0.2	0.35	-	134	-
[45], 2019	CPSGS	0.6950	-	0.85	-	6	-
[46], 2020	CPSGS	1.5882	$5\times 10^{-4}$	3.57	3966	-	-
[52], 2022	CPSGS	2.3070	0.83	2.26	1899	-	$5\times 10^{-5}$
This research	CPGS	1.1121	$5.92\times 10^{-7}$	6.55	4234.06	629.28	$1.54\times 10^{-5}$

**Table 4:** Geometric parameters without samples.

Case	$f_{\mathrm{SSPPs}}$	$f_{\mathrm{nature}}$	$f_{\mathrm{res}}$ (THz)		geometric (μm)						
	(THz)	(THz)	FIT	FEM	$h_{1}$	$h_{2}$	*g*	$h_{3}$	$W_{1}$	$W_{2}$	*P*
Case1	0.6	0.80	0.5973	0.5901	50	38	500	5	20	12	60
Case2	0.7		0.6957	0.6907	45	30	484				
Case3	0.8		0.8025	0.7997	40	25	437				

**Table 5:** Geometric parameters with samples.

Case	$f_{\mathrm{SSPPs}}$	$f_{\mathrm{nature}}$	$f_{\mathrm{res}}$ (THz)		geometric(μm)						
	(THz)	(THz)	FIT	FEM	$h_{1}$	$h_{2}$	*g*	$h_{3}$	$W_{1}$	$W_{2}$	*P*
Case1	0.6	0.80	0.4286	0.4260	50	38	677	5	20	12	60
Case2	0.7		0.5186	0.5153	45	30	559				
Case3	0.8		0.5710	0.5662	40	25	547				

## Data Availability

Not applicable.
